# Ultrasound-Assisted Recovery of Pectic Fractions from Grape Pomace: Combined Effects of Acid Type, pH, and Temperature on Physicochemical and Antioxidant Properties

**DOI:** 10.3390/polym18202465

**Published:** 2026-10-09

**Authors:** Mohamed Brahmi, Sara Moumnassi, Adem Gharsallaoui

**Affiliations:** LAGEPP, UMR 5007, CNRS, Université Lyon 1, 01000 Bourg-en-Bresse, France; mohamed.brahmi@univ-lyon1.fr (M.B.);

**Keywords:** grape pomace, pectin, ultrasound-assisted extraction, citric acid, hydrochloric acid, degree of esterification, molecular weight, antioxidant activity

## Abstract

Grape pomace represents an underexploited source of pectic polysaccharides whose properties are strongly influenced by extraction conditions. This study investigated the combined effects of acid type (citric acid or HCl), pH (1.5, 2.0 and 3.0), and temperature (55, 65 and 85 °C) on pectic fractions recovered from grape pomace using sequential ultrasound-assisted extraction followed by dynamic maceration. A 2 × 3 × 3 full-factorial design was applied, combining citric acid or hydrochloric acid, three pH values (1.5, 2.0 and 3.0) and three temperatures (55, 65 and 85 °C), resulting in 18 extraction conditions. The recovered fractions were characterized in terms of extraction yield, degree of esterification (DE), methoxyl content (MeO), anhydrouronic acid content (AUA), molecular weight (Mw), apparent viscosity, carbohydrate and protein contents, color, FTIR profile, total phenolic content (TPC) and antioxidant activity. Extraction yield ranged from 1.12 to 7.88%, with the highest recovery obtained using citric acid at pH 3.0 and 55 °C. DE varied markedly from 14.89 to 63.63%, with citric acid consistently yielding low-methoxyl fractions under all conditions, whereas HCl at pH 1.5 yielded high-methoxyl fractions. MeO varied accordingly from 1.66 to 7.65%, while AUA remained within a comparatively narrow range of 63.27–69.24%, close to that measured for commercial citrus pectin (67.21%). Molecular weight ranged from 22.7 to 607 kDa, and increasing extraction temperature generally reduced both Mw and apparent viscosity, indicating greater structural modification under harsher conditions. FTIR spectra confirmed the preservation of the characteristic pectic polysaccharide framework while revealing differences in the carboxyl and fingerprint regions among extraction conditions. The recovered fractions contained 180.22–310.78 mg glucose eq./g total carbohydrate and 8.56–32.83 mg BSA eq./g protein. Phenolic compounds were also retained, with TPC values of 9.50–14.29 mg GAE/g and ABTS IC_50_ values ranging from 4.26 to 10.38 mg/mL. The factorial analysis demonstrated significant interactions among acid type, pH, and temperature for the main physicochemical and functional responses. Overall, the results demonstrate that these extraction variables can be adjusted to recover grape pomace pectic fractions with distinct esterification, molecular and antioxidant profiles.

## 1. Introduction

Winemaking generates substantial quantities of grape pomace (GP), the solid residue composed mainly of skins, seeds and, to a lesser extent, stems. GP generally represents about 20–30% of the mass of processed grapes, making it one of the major solid by-products of the wine industry [1]. Its seasonal production and high organic load can create management and disposal constraints for wineries, particularly when large quantities are generated over a short harvest period. Conventional destinations include composting, animal feed and other relatively low value uses, while inappropriate disposal can contribute to environmental problems associated with the degradation of its organic matter [2]. In recent years, however, the management of winemaking residues has shifted towards higher value valorization. GP has consistently been reported to retain a broad range of valuable constituents after pressing, including phenolic compounds [3,4,5], anthocyanins [6,7], dietary fiber [8], seed oil [9] and structural polysaccharides [10,11], which have progressively shifted the perception of GP from a low-value residue to a promising raw material for applications in various fields, such as pharmaceutical [12,13], food [14], nutraceutical [15], cosmetic [16] and other value-added products [17].

Among these valuable constituents, pectin represents an interesting target for further valorization of the polysaccharide fraction of GP. Pectin is a plant-cell-wall heteropolysaccharide widely used as a gelling, thickening, stabilizing and emulsifying agent, while its biodegradability and film-forming properties have also supported its development in edible coatings, packaging and delivery systems [18]. Commercial production remains largely dependent on citrus peel and apple pomace, which has encouraged the search for alternative pectin sources among other agro-industrial residues [19]. GP is particularly relevant in this context because the pectin recovered from this matrix has already shown properties suitable for different food applications. For example, GP pectin has recently been successfully used as a prebiotic stabilizer in low-fat synbiotic yogurt, improving colloidal stability, rheological properties and probiotic survival [20], while pectin recovered from red GP has been incorporated into protein-based biodegradable films with promising mechanical and barrier properties [21]. However, pectin extracted from GP does not appear to have a single characteristic structural profile. Depending on the processing conditions, fractions with different esterification states, molecular sizes and functional behaviours have been reported. This variability is important because the technological performance of pectin is closely related to its degree of esterification (DE), molecular weight, galacturonic-acid-rich backbone and associated side chains [22]. The distinction between high- and low-methoxyl pectin is of particular relevance to potential applications. High-methoxyl fractions rely primarily on acid-sugar conditions for gel formation, whereas low-methoxyl pectins can form networks through ionic cross-linking with divalent cations. Recent GP-based studies illustrate this distinction. A high-methoxyl pectin was successfully developed for the stabilization of synbiotic yogurt gels [20], whereas ultrasound–microwave recovery from red GP produced a low-methoxyl, galacturonic-acid-rich pectin which was subsequently used for biocomposite food-packaging films [21]. Thus, the extraction process is not merely a recovery step, but an important determinant of the characteristics and potential use of the resulting pectic material.

Among the available processes, acid extraction remains one of the most established approaches for pectin solubilization, and the nature of the acid, pH and temperature can markedly influence both extraction efficiency and pectin characteristics. The study of Kliemann et al., comparing citric, hydrochloric and nitric acids during pectin extraction from passion-fruit peel, demonstrated significant effects of pH, temperature and extraction time on recovery [23]. Moreover, the relevance of the acid type (organic or mineral) is not limited to optimizing the extraction yield of pectin from the plant matrix. For instance, Luo et al. reported that acetic acid extraction of apple pomace yielded pectin with higher molecular weight, DE and galacturonic acid content than mineral-acid extraction, illustrating how acid chemistry can influence the quality of the recovered polymer [24]. More generally, comparisons of organic and mineral acids have shown that the acid selected can alter yield, DE and molecular characteristics, while the associated chemical demand and environmental burden also differ substantially. Therefore, comparing the two acids at the same nominal pH provides a common pH reference, without implying equivalent titratable acidity, ionic strength, or acid speciation.

Ultrasound-assisted extraction (UAE) has increasingly been combined with acidic media to intensify pectin recovery. Acoustic cavitation promotes disruption of the plant matrix and improves solvent penetration and mass transfer, thereby reducing the extraction time required compared with prolonged conventional heating. Beyond improving extraction efficiency, these effects can also influence the recovery and preservation of bioactive constituents from plant matrices. Ultrasound processing has been reported to affect phenolic compounds, pigments and antioxidant properties, although the response depends strongly on the matrix and treatment conditions [25]. At the same time, the collapse of cavitation bubbles generates local mechanical forces that can promote pectin-chain scission and depolymerization [26]. Such effects have been associated with decreases in molecular weight and viscosity, while the main pectic structure may remain largely preserved; changes in the degree of methylation have also been reported depending on the sonication conditions [27,28]. Similar observations have been reported during UAE of apple pomace, where acid type, temperature and treatment time affected yield, DE, methoxylation and surface charge of the recovered pectin [29]. Beyond these effects on pectic polysaccharides, ultrasound-induced disruption of plant tissues can also modify the release and preservation of other bioactive constituents. In vegetable matrices, ultrasound processing has been associated with increased extractability of phenolic compounds, flavonoids and carotenoids, whereas excessive treatment may reduce more sensitive constituents, showing that its effect on nutritional and functional properties strongly depends on treatment conditions [25]. This aspect is particularly relevant to GP, which contains substantial amounts of phenolic compounds, pigments, proteins and other carbohydrates that may be co-recovered with the pectic material during extraction and precipitation and thereby contribute to the characteristics of the resulting fraction. This possibility is increasingly being exploited rather than regarded exclusively as an impurity. Gurev et al., for example, reported co-extracted polyphenols and antioxidant activity in pectin obtained from apple pomace by ultrasound- and microwave-assisted extraction [30]. Assessing phenolic retention and antioxidant activity together with conventional pectin characteristics can therefore provide a more complete picture of the material obtained from this complex by-product.

In this context, the present study investigated the combined effects of acid type, extraction pH and temperature on pectic fractions recovered from the same batch of red grape pomace. The present work was designed to directly compare citric acid and hydrochloric acid (HCl) under the same pH and temperature conditions. A 2 × 3 × 3 full factorial design combining three pH values (1.5, 2.0 and 3.0), three temperatures (55, 65 and 85 °C) and two acids was therefore applied to a sequential extraction process involving ultrasound-assisted treatment followed by dynamic maceration. Beyond extraction yield, the resulting 18 fractions were characterized in terms of degree of esterification, methoxyl content and anhydrouronic acid content, molecular weight, apparent viscosity, carbohydrate and protein contents, colour and FTIR profile. Given the phenolic-rich nature of grape pomace, the possible co-recovery of phenolic compounds within the pectic fractions was also investigated through total phenolic content and ABTS radical-scavenging activity. This approach allowed the individual and interactive effects of acid type, pH and temperature to be examined across both the physicochemical characteristics of the recovered pectic fractions and their associated antioxidant properties.

## 2. Materials and Methods

### 2.1. Grape Pomace Collection and Pretreatments

The winemaking by-product was supplied by a local winery (Frères Castel, Saint-Priest, France) as a concentrated residue derived from a blend of red grape varieties comprising Merlot, Syrah, Grenache, and Tempranillo. The as-received material contained approximately 30% solids, as determined gravimetrically after drying. The material was air-dried for 48 h under a laboratory fume hood at room temperature with limited exposure to light and subsequently ground using a laboratory grinder to obtain a fine powder with an approximate particle size of 20 µm. After freeze-drying, the residual moisture content of the prepared grape-pomace powder was 3.45 ± 0.80%. The powder was stored at −20 °C until further use.

### 2.2. Sequential Ultrasound-Assisted Extraction and Dynamic Maceration for the Recovery of Pectic Fractions from Grape Pomace

Pectic fractions were recovered from GP using sequential ultrasound-assisted extraction followed by dynamic maceration, adapted from the combined ultrasonication/maceration approach described by [31], with modifications for the recovery of pectic fractions from grape pomace.

A full-factorial experimental design was used to investigate the effects of acid type, extraction pH, and temperature. Two acidifying agents were evaluated, namely citric acid and HCl, at three pH values (1.5, 2.0, and 3.0) and three extraction temperatures (55, 65, and 85 °C), resulting in 18 extraction conditions. A solid-to-liquid ratio of 1:20 (*w*/*v*), selected from preliminary yield experiments in which ratios of 1:10, 1:20, 1:50, 1:80, and 1:100 (*w*/*v*) were compared under a fixed extraction condition, was maintained for all extraction conditions.

GP powder was dispersed in distilled water at a solid-to-liquid ratio of 1:20 (*w*/*v*), and the extraction medium was adjusted to pH 1.5, 2.0, or 3.0 using either 1 M citric acid or 1 M HCl.

The suspensions were transferred to glass flasks and subjected to ultrasound treatment for 20 min using an ultrasonic bath (TUC-100, Dongguan, China) operated at 40 kHz and 240 W (100% power), while maintaining the corresponding extraction temperature. Before ultrasonication, the bath was preheated to the target extraction temperature (55, 65, or 85 °C). The bath temperature was maintained throughout the treatment using the instrument temperature control, while ice was added when necessary to counteract the temperature increase associated with ultrasonic treatment. Under these conditions, the observed temperature variation remained within approximately +2 °C of the target value. Immediately after ultrasonication, the suspensions were subjected to dynamic maceration under continuous stirring at 900 rpm for 2 h while maintaining the same temperature.

At the end of extraction, insoluble residues were removed by centrifugation (OHAUS, Frontier 5000 Multi-Pro, Nänikon, Switzerland) at 9000 rpm for 10 min. The supernatants containing solubilized pectic material were collected and mixed with 96% (*v*/*v*) ethanol at an extract-to-ethanol ratio of 1:3 *v*/*v*. The mixtures were maintained at 4 °C overnight to allow complete precipitation. The precipitated pectic fraction was subsequently recovered and washed twice with 96% ethanol. For further purification, the recovered material was re-dissolved in distilled water and re-precipitated with 96% ethanol, and this dissolution–precipitation step was repeated before final recovery. The recovered samples were frozen at −20 °C overnight and subsequently freeze-dried using a Lyovapor™ L-200 freeze-dryer (Lyovapor™ L-200, Büchi, Rungis, France) for 48 h under a chamber pressure of 0.08 mbar and a condenser temperature of −54 ± 0.5 °C. Each extraction condition was independently performed in triplicate. The visualization of the extraction procedure is illustrated in Figure 1.

### 2.3. Determination of the Extraction Yield

The extraction yield was determined on a dry-weight basis as the mass of freeze-dried pectic fraction recovered relative to the initial dry mass of GP used for extraction (1).

(1) $$\mathrm{Yield}(\%)=\frac{m_{\mathrm{Pectin}}}{m_{\mathrm{GP}}}\times 100$$

where *m_Pectin_* is the mass of recovered freeze-dried sample (g) and *m_GP_* is the initial dry mass of GP powder used for extraction (g).

### 2.4. Characterization of the Extracted Pectic Fraction

#### 2.4.1. Degree of Esterification

The degree of esterification (DE) of the extracted samples and commercial citrus pectin (CCP; Sigma-Aldrich, St-Quentin Fallavier, France), used as an external reference, was determined using a titrimetric method adapted from [32]. In brief, 0.2 g of the extract was dispersed in 25 mL of distilled water and maintained at 50 °C until complete hydration and dissolution. Four drops of phenolphthalein indicator were subsequently added. The solution was first titrated with 0.25 M NaOH until a persistent pale-pink endpoint was obtained. The volume of NaOH consumed during this first titration was recorded as *V*_1_ and corresponded to neutralization of free carboxyl groups. Subsequently, 10 mL of 0.25 M NaOH was added, and the solution was stirred for 30 min to hydrolyse the esterified carboxyl groups. Thereafter, 10 mL of 0.25 M HCl was added and the solution was mixed until the pink coloration disappeared. The remaining acid was titrated with 0.10 M NaOH until the pale-pink endpoint was restored, and the corresponding volume was recorded as *V*_2_. The DE (%) was accordingly calculated on a molar-equivalent basis as follows (2):

(2) $$\mathrm{DE}\left(\%\right)=\frac{C_{2}V_{2}}{C_{1}V_{1}+C_{2}V_{2}}\times 100$$

where *V*_1_ and *V*_2_ are the NaOH volumes used during the first and second titrations, respectively, and *C*_1_ and *C*_2_ are their corresponding molar concentrations.

#### 2.4.2. Determination of Methoxyl and Anhydrouronic Acid Contents

Methoxyl content (MeO) and anhydrouronic acid content (AUA) of the extracted pectic fractions and CCP were determined by titration according to the method described by [33]. The pectic fraction was dispersed in deionized water after moistening with ethanol and addition of sodium chloride. Phenolphthalein was used as the endpoint indicator. The suspension was first titrated with 0.1 N NaOH to neutralize the free carboxyl groups, and the consumed volume was recorded as *V*_1_. The solution was then treated with 0.25 N NaOH and maintained at room temperature for 30 min to saponify the esterified carboxyl groups. An equivalent volume of 0.25 N HCl was subsequently added, and the released carboxyl groups were titrated with 0.1 N NaOH; this second volume was recorded as *V*_2_.

The methoxyl content was calculated as:

(3) $$\mathrm{MeO}\left(\%\right)=\frac{V_{2}\times N\times 31\times 100}{m\times 1000}$$

The anhydrouronic acid content was calculated from the total milliequivalents consumed in the two titration steps:

(4) $$\mathrm{AUA}\left(\%\right)=\frac{176\times N\times \left(V_{1}+\;V_{2}\right)\times 100}{m\times 1000}$$

where *V*_1_ and *V*_2_ are the volumes of NaOH consumed during the first and second titrations, respectively, N is the normality of NaOH, m is the sample mass (g), 31 g/mol corresponds to the molar mass of the methoxyl group and 176 g/mol is the molar mass of the anhydrogalacturonic acid unit.

#### 2.4.3. Molecular Weight (M_w_)

The molecular weight M_w_ of the extracted pectic fractions and CCP was determined by static light scattering (SLS) using a Zetasizer Nano ZS90 (Malvern Instruments Ltd., Worcestershire, UK) operating at a fixed scattering angle of 90°. Prior to analysis, pectic fraction samples were fully hydrated and solubilized in distilled water at 50 °C for 4 h, then centrifuged at 8000 rpm for 5 min before analysis. Four concentrations (0.05, 0.06, 0.08, and 0.10 mg/mL) were subsequently prepared from each sample. A refractive index increment (dn/dc) of 0.150 mL/g was used for molecular weight calculations. Water was used as the solvent (RI = 1.330 at 25 °C), while toluene was used as the scattering reference (RI = 1.496; Rayleigh ratio = 1.35 × 10^−5^ cm^−1^). Molecular-weight determination was based on the concentration dependence of the Rayleigh ratio according to the Debye relationship (5):

(5) $$\frac{\mathrm{Kc}}{R_{\theta }}=\frac{1}{M_{w}}+2A_{2}c$$

where *c* is the polymer concentration, *R_θ_* is the Rayleigh ratio, *K* is the optical constant, *M_w_* is the molecular weight, and *A*_2_ is the second virial coefficient. The *M_w_* value was obtained from the intercept of the Debye plot generated by the instrument software (Zetasizer Software, version 7.10, Malvern Instruments Ltd., Worcestershire, UK). Measurements were performed in triplicate, and results were expressed in kDa.

#### 2.4.4. FTIR Measurements

The chemical functional groups of the 18 extracted samples were characterized by attenuated total reflectance Fourier-transform infrared (ATR-FTIR) spectroscopy using an IRSpirit FTIR spectrometer (Shimadzu, Kyoto, Japan) equipped with a QATR™-S single-reflection ATR accessory containing a diamond crystal (Shimadzu, Kyoto, Japan). Spectra were collected over the range 4000–500 cm^−1^ at a spectral resolution of 8 cm^−1^, with 64 scans per spectrum. Commercial citrus pectin (Sigma-Aldrich) was analyzed under the same conditions and used as a reference for spectral comparison.

#### 2.4.5. Apparent Viscosity

The apparent viscosity of the recovered pectic fractions and CCP (2%, *w*/*v*, in deionized water) was measured using a Brookfield DVNext viscometer (AMETEK Brookfield, Élancourt, France) equipped with an LV-02 spindle (No. 62) operating at 20 rpm. Measurements were performed at 25 ± 1 °C, with the temperature monitored using the sensor connected to the instrument. Measurements were carried out in triplicate and expressed in mPa·s.

#### 2.4.6. Color Measurements

The color characteristics of the freeze-dried pectic fraction powders and CCP were determined using a CM-25cG portable spectrophotometer (Konica Minolta, Tokyo, Japan). Samples were placed in identical transparent Petri dishes during measurement, and measurements were performed under identical instrumental conditions. Color coordinates were expressed according to the CIE L^*^ a^*^ b^*^ color space, where L^*^ represents lightness, a^*^ represents the red-green coordinate, and b^*^ represents the yellow–blue coordinate. The total color difference (ΔE*) between each extracted pectic fraction and the selected reference was calculated as:

(6) $${\Delta E}^{*}=\sqrt{{\left(L^{*}-L_{0}^{*}\right)}^{2}+{\left(a^{*}-a_{0}^{*}\right)}^{2}+{\left(b^{*}-b_{0}^{*}\right)}^{2}}$$

where $L_{0}^{*}$, $a_{0}^{*}$ and $b_{0}^{*}$ are the color coordinate of the reference. All measurements were performed in triplicate, and results are reported as mean ± standard deviation.

#### 2.4.7. Determination of Total Carbohydrate and Protein Contents

Total carbohydrate content of the recovered fractions and CCP was determined using the phenol–sulfuric acid colorimetric method described by DuBois et al. [34]. Glucose was used to construct the calibration curve, and carbohydrate contents were expressed as milligrams of glucose equivalents per gram of the extract (mg glucose eq./g pectic fraction).

Protein content of the recovered fractions and CCP was determined using the Bradford dye-binding method [35]. Bovine serum albumin (BSA) was used as the calibration standard, and results were expressed as milligrams of BSA equivalents per gram of extracted pectic fraction (mg BSA eq./g pectic fraction).

### 2.5. Determination of Total Phenolic Content (TPC)

To investigate the possible co-extraction of phenolic compounds with the pectic polysaccharide matrix, the total phenolic content (TPC) of the recovered pectic fractions was determined using the Folin–Ciocalteu assay as a function of the extraction conditions, and CCP was analysed under the same conditions as a commercial reference. Solutions of each recovered pectic fraction (P1–P18) and CCP were prepared in distilled water at 25 mg/mL. The solutions were heated at 70 °C for 2 h under continuous agitation at 900 rpm to ensure complete solubilization.

After complete solubilization, TPC was determined using the Folin–Ciocalteu colorimetric method. Briefly, 10 µL of each solution was mixed with 100 µL of distilled water, followed by the addition of 40 µL of Folin–Ciocalteu reagent previously diluted 1:9 (*v*/*v*) with distilled water. The mixture was incubated for 4 min before the addition of 150 µL of a 20% (*w*/*v*) sodium carbonate solution. The reaction mixture was then incubated for 2 h at room temperature in the dark. Absorbance was measured at 760 nm using a microplate reader. TPC was calculated from a gallic acid calibration curve and expressed as milligrams of gallic acid equivalents per gram of dry pectic fraction (mg GAE/g dry pectic fraction).

### 2.6. Antioxidant Activity of the Recovered Pectic Fractions

The antioxidant activity of the extracted pectic fractions (P1–P18) and of CCP, used as a commercial reference, was evaluated using the ABTS radical cation scavenging assay. The ABTS•+ radical cation was generated by mixing equal volumes of a 7.0 mM ABTS solution and a 2.45 mM potassium persulfate solution. The mixture was incubated in the dark at 25 °C for 16 h to allow radical formation. Before use, the ABTS•+ solution was diluted with Milli-Q water to obtain an absorbance of 0.70 ± 0.015 at 734 nm.

The assay was performed in a 96-well microplate. Briefly, 30 µL of pectic fraction solution at concentrations ranging from 1 to 25 mg/mL was mixed with 270 µL of the diluted ABTS•+ solution. The reaction mixture was incubated for 6 min, after which the absorbance was measured at 734 nm. The radical scavenging activity was calculated as the percentage inhibition relative to the control. The IC_50_ value, defined as the concentration of pectic fraction required to inhibit 50% of the ABTS•+ radicals, was determined from the concentration–inhibition curve and expressed in mg/mL.

### 2.7. Statistical Analysis

All extractions were performed as three independent replicates (*n* = 3), each starting from a separate portion of GP powder and carried through the complete extraction, precipitation and freeze-drying sequence. Results are therefore expressed as the mean ± standard deviation of three independent extractions. The effects of acid type, extraction pH, and temperature on the measured responses were evaluated using a three-way factorial analysis of variance (ANOVA). The model included the three main effects as well as the acid × pH, acid × temperature, pH × temperature, and acid × pH × temperature interaction terms. Because the influence of temperature was also examined within each individual acid–pH condition, pairwise comparisons among the three extraction temperatures were performed using the Games–Howell test, which does not assume homogeneity of variances. Differences were considered statistically significant at *p* < 0.05. Statistical analyses were conducted in Python (version 3.13.5) using the SciPy and statsmodels statistical packages.

## 3. Results and Discussion

### 3.1. Factorial Analysis of Extraction Variables

The effects of acid type, extraction pH, and temperature on the recovery and physicochemical characteristics of GP pectic fractions were first evaluated using a three-way factorial ANOVA. The analysis was intended to distinguish the individual contribution of each extraction factor from the interactions among them, since the response to one variable may depend on the levels of the other two. The resulting significance patterns are summarized in Table 1, while the complete ANOVA outputs, including degrees of freedom, sums of squares, mean squares, F values, exact *p*-values, and partial η^2^ effect sizes, are provided in Table S1 of the Supplementary Materials.

### 3.2. Extraction Yield

The extraction conditions produced marked differences in the recovery of pectic material from grape pomace, with yields ranging from 1.12 ± 0.67 to 7.88 ± 0.15% on a dry-weight basis (Table 2). The highest yield was obtained with citric acid at pH 3.0 and 55 °C (P1), whereas the lowest was recorded with HCl at pH 2.0 and 65 °C (P14).

The factorial analysis confirmed significant effects of acid type, pH and temperature, but also significant acid × temperature, pH × temperature and acid × pH × temperature interactions (Table 1). These interactions are reflected directly in the experimental trends. At pH 3.0 and 2.0, 55 °C consistently gave the highest recovery for both acids. In contrast, at pH 1.5 the maximum shifted towards higher temperatures, whereas citric acid extraction increased from 2.00% at 55 °C to approximately 3.2% at 65–85 °C, while the highest HCl yield at this pH was obtained at 85 °C. Thus, temperature did not exert a uniform effect on the recovery of pectic material, but its influence depended strongly on the acidity of the extraction medium. Moreover, citric acid consistently resulted in higher extraction yields than HCl, with the difference being particularly pronounced at the two lower pH values. This trend is consistent with previous studies conducted on different plant matrices [36,37]. Previous reports have linked the higher extraction efficiency of citric acid, at least in part, to its chelating properties in addition to its acidifying effect. In particular, the complexation of Ca^2+^ by citric acid has been proposed to weaken calcium-mediated associations within pectic domains of the plant cell wall, thereby facilitating the solubilization and release of pectic material [38,39]. This mechanism therefore represents one possible explanation for the greater recovery observed with citric acid under the present extraction conditions. It should also be considered that adjustment to the same nominal pH does not imply equivalent acidification conditions. Citric acid and HCl differ markedly in their dissociation behaviour, and previous pectin-extraction studies have shown that substantially different amounts of organic and mineral acids may be required to establish the same extraction pH [40]. Consequently, the effect associated with acid type may reflect not only proton concentration, but also differences in the chemical environment generated by the respective acid systems.

This behavior is compatible with the competing processes involved in acidic extraction. Increasing temperature can enhance solvent penetration, mass transfer and disruption of interactions retaining pectic polysaccharides within the cell wall, and several grape pomace-related studies have consequently reported higher yields at elevated temperatures and low pH. Spinei and Oroian, for example, observed an increase in yield from 4.23 to 7.30% between 70 and 90 °C at pH 2, and also reported greater recovery as pH decreased [41]. Minjares-Fuentes et al. likewise reported a maximum yield of pectin recovery at 75 °C and pH 2.0, using citric acid-assisted ultrasound extraction [42]. However, this trend is clearly not universal. Vakilian et al. recently identified optimum UAE conditions for ripe GP at pH 2.99 and 58.82 °C, remarkably close to the region producing the greatest recovery in the present work [43].

A further consideration is that the gravimetric yield represents the total material recovered after ethanol precipitation, rather than chemically pure pectin alone. Ethanol precipitation is not completely selective; Garna et al. demonstrated that non-pectic compounds released from pomace can co-precipitate with pectic material and thereby contribute to apparent extraction yield [44]. This point is particularly relevant for grape pomace, which contains abundant phenolic compounds and can yield pectin–polyphenol or pectin–protein–polyphenol assemblies during extraction [45], as the mild temperature and acidity conditions were repeatedly reported as favorable conditions for polyphenol recovery from grape pomace [46].

### 3.3. Degree of Esterification, Methoxyl Content and Anhydrouronic Acid Content

The esterification-related characteristics of the recovered pectic fractions were assessed through their anhydrouronic acid content (AUA), methoxyl content (MeO), and degree of esterification (DE) (Table 3). AUA ranged from 63.27 to 69.24%, with most fractions showing values close to that of the commercial citrus pectin (CCP, 67.21%). These results support the substantial contribution of uronic-acid-rich pectic material to the recovered fractions. The factorial analysis showed that AUA was significantly affected by acid type and temperature (*p* < 0.001), whereas the main effect of pH was not significant. Significant two-way interactions were also observed, although the three-way acid × pH × temperature interaction was not significant. Despite these statistical effects, the numerical variation in AUA remained relatively limited. The clearest temperature-dependent change was observed for citric acid at pH 3.0, where AUA increased significantly from 63.27% at 55 °C to 68.40 and 69.24% at 65 and 85 °C, respectively, while no significant temperature-related differences were observed within the other acid–pH combinations. Comparable values have been reported for grape-pomace pectic material, including 63.5% uronic acids in a purified Chardonnay grape-pomace fraction [47].

Greater variation was observed for MeO and DE, which ranged from 1.66 to 7.65% and from 14.89 to 63.63%, respectively. The highest values were obtained under HCl extraction at pH 1.5 and approached those measured for CCP, which showed a MeO content of 8.64% and a DE of 72.95%. The factorial analysis showed significant effects of acid type, pH, and temperature on both MeO and DE (*p* < 0.001), together with significant interaction effects, including the three-way interaction (*p* < 0.001). This confirms that the esterification characteristics of the recovered fractions depended on the combined extraction conditions rather than on any single factor.

For citric-acid extraction, all fractions remained within the low-methoxyl range (DE < 50%). At pH 3.0, both DE and MeO remained relatively stable across the three temperatures. At pH 2.0, DE increased significantly at 85 °C, from 22–23% at 55–65 °C to 30.00%, while MeO similarly increased to 3.55%. The most pronounced temperature effect was observed at pH 1.5, where DE increased from 14.89% at 55 °C to 34.48% at 65 °C and 43.47% at 85 °C, accompanied by a rise in MeO from 1.66 to 3.95 and 5.00%, respectively. This behavior illustrates the strong interaction between pH and temperature observed in the factorial analysis.

A different profile was observed with HCl. At pH 2.0 and 3.0, the recovered fractions remained low-methoxyl, with DE values between 23.52 and 33.33%. At pH 3.0, DE increased significantly only at 85 °C, while MeO increased progressively from 2.69 to 3.61%. At pH 2.0, DE increased from 25.00% at 55 °C to 33.33% at 85 °C, accompanied by an increase in MeO from 2.94 to 4.02%. In contrast, HCl extraction at pH 1.5 yielded high-methoxyl fractions, with DE values of 57.14–63.63% and MeO contents of 6.83–7.65%. The significant acid × pH and acid × temperature interactions (*p* < 0.001) therefore reflect the markedly different esterification profiles obtained with the two acid systems under otherwise comparable extraction conditions.

The broad range of esterification states observed here agrees with the considerable variability previously reported for grape-pomace pectin. Minjares-Fuentes et al. reported a DE of 55.2% after citric-acid-assisted ultrasound extraction at pH 2.0 and 75 °C [42], whereas Colodel et al. obtained a degree of methyl esterification of 18.1% for a Chardonnay grape-pomace pectic fraction extracted using nitric acid [47]. In contrast, Spinei and Oroian reported DE values of approximately 80–81% under optimized conventional and pulsed ultrasound-assisted extraction conditions [48]. These reports are consistent with the present factorial results and indicate that the esterification characteristics of grape-pomace pectic material arise from the combined extraction environment rather than from the isolated effect of acid type, pH, or temperature.

### 3.4. FTIR Characterization

ATR-FTIR spectroscopy was used to further examine the structural characteristics of the extraction fractions. For clarity, Figure 2a–e present the spectra of the fractions obtained at 85 °C, allowing the effects of acid type and pH to be compared while keeping the extraction temperature constant. The complete ATR-FTIR spectra of all 18 recovered fractions are provided in the Supplementary Materials (Figure S1), allowing the effect of temperature to be visualized within each acid–pH condition.

The spectra showed that all extracted samples retained a structural profile characteristic of pectic polysaccharides, while noticeable differences from commercial citrus pectin and among the extraction conditions indicated changes in the chemical environment of the recovered material. All spectra exhibited the broad O-H stretching region around 3310–3266 cm^−1^, together with C-H stretching bands around 2919–2850 cm^−1^. These bands are characteristic of polysaccharides and reflect hydroxyl-rich structures and the CH, CH_2_ and CH_3_ groups of the carbohydrate backbone.

In the extracted samples, slight changes in the position and broadness of the O-H region compared with commercial citrus pectin suggest differences in hydrogen-bonding environments. Such modifications have also been observed in grape-pomace pectin containing associated polyphenols and proteins, where interactions with these components altered the O-H and C-H regions relative to commercial pectin [45].

More pronounced differences were visible in the carbonyl and carboxyl region (Figure 2c–e). Commercial citrus pectin showed the expected bands around 1735 and 1610 cm^−1^, whereas the grape-pomace extracts displayed carbonyl bands shifted toward approximately 1707–1727 cm^−1^, together with absorptions around 1635–1640 cm^−1^. The first region is associated mainly with carbonyl-containing carboxylic and ester groups, whereas the latter includes contributions from ionized carboxylate groups. The variation between these regions is coherent with the differences in DE reported above. However, in the case of pectin extracted from GP matrix, these bands cannot be attributed exclusively to pectin esterification. For instance, previous FTIR studies on pectin recovered from GP have shown that absorptions around 1600 cm^−1^ may also include aromatic C=C vibrations from associated phenolic compounds, while nearby bands can contain contributions from protein amide groups [49]. The shifts observed relative to commercial citrus pectin may therefore reflect not only differences in esterification, but also a different local environment of the carboxyl groups resulting from hydrogen bonding and association with other grape-derived constituents.

The characteristic carbohydrate fingerprint region (1200–850 cm^−1^) was also preserved across the different extraction conditions. The bands within this range are commonly associated with C–O, C–C, and C–O–C vibrations of glycosidic linkages and pyranose-ring structures in pectic polysaccharides [50]. Overall, considering that the present extracts also contained measurable amounts of proteins and phenolic compounds, the observed spectral differences are consistent with the recovery of a more compositionally complex grape-pomace pectic fraction rather than a highly purified polysaccharide.

### 3.5. Molecular Weight and Apparent Viscosity

The molecular weight (M_w_) of the recovered pectic fractions showed a strong dependence on the extraction conditions, ranging from 22.7 to 607 kDa (Figure 3a). The highest values were mainly obtained at pH 2.0, especially at the lower extraction temperatures, whereas increasing temperature generally reduced M_w_. For pectic fractions extracted using citric acid, M_w_ decreased from 110.0 to 34.1 kDa at pH 3.0 and from 137.3 to 36.7 kDa at pH 1.5 between 55 and 85 °C (*p* < 0.05). At pH 2.0, much higher values were obtained at 55–65 °C (537–607 kDa), followed by a significant decrease at 85 °C (*p* < 0.05). A similar effect was observed with HCl-based extraction, particularly at pH 3.0, where M_w_ decreased to 22.7 kDa at 85 °C. These results are consistent with the well-established sensitivity of pectin chains to acidic and thermal conditions. Thermal treatment under acidic conditions promotes the hydrolysis of glycosidic linkages, consequently decreasing the molecular weight of pectin. This behavior has been demonstrated experimentally during controlled acid hydrolysis of pectin [51]. Consistent with this trend, Yoo et al. reported that pectin extracted by microwave-assisted extraction at pH 2 exhibited a molecular weight more than fivefold higher than that of pectin extracted at pH 1 [52].

The apparent viscosity generally decreased as extraction temperature increased (Figure 3b), following the same overall tendency observed for molecular weight. In most series, increasing extraction temperature significantly reduced viscosity (*p* < 0.05), particularly for the citric acid-based samples. At pH 1.5, for example, viscosity decreased from 45.6 to 31.0 mPa·s, while at pH 3.0 it decreased from 19.2 to 14.4 mPa·s. This behavior is consistent with the established relationship between pectin molecular size and viscosity, since shorter polymer chains have a lower hydrodynamic contribution in solution [53]. Acid hydrolysis has also been shown experimentally to cause simultaneous decreases in pectin molecular weight and viscosity [51]. However, this relationship was not strictly proportional for all samples. P7, for example, exhibited the highest viscosity despite having a substantially lower M_w_ than several pH 2.0 samples, indicating that viscosity was not governed by molecular weight alone. In addition to molecular weight, hydrodynamic behavior is also influenced by structural characteristics such as degree of esterification and chain conformation. Morris et al., using citrus pectin of comparable molecular weight (190,000 ± 30,000 g/mol) but different DE values, demonstrated that esterification can alter intrinsic viscosity and chain stiffness [54]. More generally, the rheological behavior of pectin is determined by the combined influence of molecular size and structural characteristics such as esterification and molecular organization [55]. Overall, the decrease in both M_w_ and viscosity observed in most extraction series indicates progressive modification of the pectic chains as extraction temperature increased. For comparison, CCP showed an Mw of 101.23 ± 4.15 kDa and an apparent viscosity of 25.01 ± 0.98 mPa·s, both of which fall within the ranges obtained for the grape-pomace pectic fractions.

### 3.6. Carbohydrate and Protein Content of the Extracts

The effects of pH, acid type and temperature on the composition of the recovered pectic fractions were subsequently evaluated in terms of carbohydrate and protein content. Figure 4 shows the values obtained for each sample. Total carbohydrate content (Figure 4a) ranged from 180.22 ± 7.04 to 310.78 ± 3.05 mg glucose eq./g pectic fraction, whereas CCP, analysed under the same conditions, showed a considerably higher value (698.15 ± 2.15 mg glucose eq./g). Acid type alone had no significant overall effect, whereas pH and temperature significantly influenced the response (Table 1). The highest values were generally obtained at pH 1.5 and 2.0, where most samples contained approximately 300 mg glucose eq./g. At pH 3.0, lower values were observed, particularly for the HCl series, where carbohydrate content decreased significantly from 260.89 to 180.22 mg/g between 55 and 85 °C (*p* < 0.05). A similar trend was observed for the citric acid series at pH 3.0, which gave the lowest carbohydrate contents overall. This supports the interpretation proposed above, namely that the higher yields recorded at pH 3.0 should be treated with caution, since non-polymeric compounds may be co-extracted under these milder conditions. By contrast, lowering the pH gave higher and comparable carbohydrate contents for both acids. This suggests that a lower pH combined with a higher temperature may alter the molecular weight and rheological properties through fragmentation of the polymeric chains, while leaving the total carbohydrate content essentially unchanged.

These values should, however, be read as total carbohydrate expressed in glucose equivalents rather than as a measure of pectin purity. The phenol–sulfuric acid assay responds differently to the various sugars present in complex polysaccharides, and uronic acids, which constitute the main backbone of pectin, produce a considerably lower molar response than neutral sugars. Consequently, glucose-equivalent values cannot be directly converted into galacturonic-acid content or percentage pectin [56]. For example, Colodel et al. recovered a pectic fraction containing approximately 25% glucose when extracting pectin from grape pomace; part of this glucose was subsequently identified as starch and removed enzymatically before detailed characterization. Their final purified fraction contained 63.5% uronic acids [47], which illustrates that not all of the carbohydrate co-recovered during GP extraction belongs to the pectic backbone.

Protein content showed a different pattern, ranging from 8.56 ± 4.95 to 32.83 ± 2.06 mg BSA eq./g pectic fraction, corresponding to approximately 0.9–3.3% (*w*/*w*), while CCP contained only 1.15 ± 0.13 mg BSA eq./g. As observed for total carbohydrate, acid type alone had no significant overall effect, whereas pH significantly influenced protein recovery (Table 1). Temperature effects within individual acid–pH groups were generally limited, although some condition-specific differences were observed. The presence of measurable protein in the recovered grape-pomace pectic fractions is consistent with previous reports. For instance, grape pomace pectin obtained using different food-grade extraction procedures contained 1.3–5.4% protein, including approximately 2.2% in the citric acid-extracted fraction [57]. More recently, a white-grape-pomace pectic preparation containing 10.4% protein and 68.2% uronic acids was characterized as a pectin–protein–polyphenol complex, showing that proteinaceous material can remain associated with grape-derived pectin after extraction and precipitation [45]. The presence of appreciable amounts of protein in the recovered pectic fractions under the various conditions tested may add functional value to the extracted fraction, since the emulsifying properties of pectin are largely attributed to hydrophobic moieties of protein bound to the carbohydrate backbone [58].

### 3.7. Color Characteristics

The extracted samples showed noticeable differences in color depending mainly on the extraction pH (Table 4). A clear tendency toward darker and redder powders was observed as pH decreased. Mean lightness (L*) decreased from approximately 67.7 at pH 3.0 to 62.0 at pH 2.0 and 57.6 at pH 1.5, whereas redness (a*) increased from 10.2 to 13.8 and 16.0, respectively. The lightest sample was P1 (L* = 73.28), while the lowest L* values were obtained for the citric acid-based samples at pH 1.5. HCl extracts were also generally redder than the corresponding citric acid samples. In contrast, temperature did not produce a consistent monotonic effect on the color parameters across the different acid–pH combinations.

This behavior is particularly relevant for pectic fractions obtained from red grape pomace, since pigments and other phenolic compounds can remain associated with the recovered polysaccharide during extraction and precipitation. Accordingly, CCP showed a lighter and considerably less red color profile (L* = 76.30 ± 0.01; a* = 3.65 ± 0.00; b* = 15.10 ± 0.01) than the recovered grape-pomace fractions. Previous studies have similarly reported lower lightness and reddish coloration compared with commercial pectins and attributed these characteristics mainly to the retention of anthocyanins and tannins [49]. The stronger redness observed at low pH is also consistent with anthocyanin chemistry. Anthocyanins from red grape skins are predominantly present as the intensely red flavylium cation under strongly acidic conditions, particularly below pH 2, whereas increasing pH promotes conversion toward less intensely colored forms [59]. More recent work on valorization of GP also confirms that acidified aqueous media are suitable for recovering anthocyanins and that their stability is favored in the strongly acidic range [7]. Taken together, the color differences recorded here are consistent with variations in the amount and chemical form of the grape-derived pigments carried through pectic fraction recovery. The pigments predominant in red grape pomace, mainly anthocyanins and tannins, are phenolic in nature, and the following sections therefore quantify the total polyphenol content of the extracts and evaluate the antioxidant activity of the extracted pectic fractions.

### 3.8. Total Phenolic Content and Antioxidant Activity

Analysis of the total phenolic content showed that the pectic fractions retained appreciable amounts of phenolic compounds, with TPC ranging from 9.50 ± 0.24 to 14.29 ± 0.35 mg GAE/g, markedly higher than that of CCP (1.01 ± 0.15 mg GAE/g), while ABTS IC_50_ values ranged from 4.26 ± 0.14 to 10.38 ± 0.06 mg/mL (Table 5). The three-way ANOVA showed significant effects of acid type, pH and temperature on both responses, together with significant interactions among these factors (Table 1), confirming that phenolic retention and antioxidant activity were strongly dependent on the complete extraction environment rather than on a single variable.

A particularly consistent temperature response was observed for the citric acid-based extracts. At each pH, extraction performed at 65 °C produced the lowest ABTS IC_50_ value, indicating the strongest radical-scavenging activity within the corresponding series. The TPC results followed a similar tendency; 65 °C gave the highest value at pH 3.0 and pH 1.5, while at pH 2.0 the values obtained at 65 and 85 °C were statistically comparable (*p* > 0.05). This behavior suggests that an intermediate temperature favored the release and preservation of phenolic compounds associated with the recovered pectic fraction using citric acid. Temperature is known to enhance diffusion and mass transfer during GP extraction; however, excessive heating can also promote degradation or transformation of thermolabile phenolics. González-Centeno et al. reported simultaneous increases in phenolic recovery and antioxidant capacity as temperature increased during ultrasound-assisted extraction of GP [60], whereas more recent optimization work identified 65 °C as the optimum temperature for maximizing phenolics, anthocyanins and ABTS/DPPH activity [61].

The HCl-derived fractions showed a markedly different response. Their TPC values remained within a relatively narrow range, yet their ABTS IC_50_ values were consistently lower than those of the citric acid-based fractions, indicating stronger antioxidant activity. Such behavior is consistent with the complex effect of strong acidic conditions on grape phenolics. Hydrochloric-acid hydrolysis can release phenolic compounds from bound or conjugated forms while simultaneously modifying their composition. Martins et al. demonstrated that temperature and HCl concentration strongly affected the profile of non-extractable grape phenolics, with intermediate conditions favoring proanthocyanidins and more severe hydrolysis favoring hydrolysable tannins and hydroxybenzoic acids. In that work, acid treatment determined not only the quantity of phenolics recovered but also the relative abundance of the individual phenolic classes [62]. Similarly, HCl hydrolysis of GP extracts has been shown to convert conjugated phenolics into their corresponding aglycones and to substantially alter the chromatographic profile as hydrolysis temperature increases [63]. Thus, the stronger antioxidant activity of the HCl-based fractions, despite their comparatively moderate TPC, may reflect the recovery or formation of phenolic species with greater radical-scavenging efficiency. Overall, the present results indicate that citric acid extraction exhibited a well-defined favorable temperature around 65 °C, whereas HCl generated pectic fractions with stronger antioxidant activity whose response was more dependent on the specific pH–temperature combination and on the composition of the co-recovered phenolic fraction.

### 3.9. Integrative Effect of Extraction Conditions on the Recovered Pectic Fractions

Taken together, the results show that the extraction conditions did not simply determine how much material was recovered from grape pomace, but also strongly influenced the characteristics of the resulting pectic fraction. Acid extraction represents an initial step in determining the properties of grape pomace pectic fractions. This process occurs alongside alterations in the extracted polymers and the recovery of other grape constituents. Consequently, extraction procedures can influence both the pectic structure and the functional properties of the recovered fraction. In the present study, the significant acid × pH × temperature interaction for most measured responses (Table 1) demonstrates that these changes depended on the combined extraction conditions. For instance, the influence of acidification varied with temperature and acid identity, explaining why adjusting the extraction conditions produced different combinations of esterification, molecular characteristics and antioxidant activity from the same raw material. This was particularly evident for DE and MeO, which varied widely across the extraction conditions, whereas AUA remained within a comparatively narrow range of 63.27–69.24%, close to the value measured for commercial citrus pectin (67.21%). This indicates that the extraction conditions markedly affected the methylation state of the recovered pectic material while its AUA content remained comparatively stable. Moreover, the conditions giving the highest extraction yield were not necessarily those yielding the largest macromolecular structures. In particular, pH 3.0 combined with 55 °C favored mass recovery, whereas the highest molecular weights were mainly observed at pH 2.0 at lower extraction temperatures. On the other hand, increasing the extraction temperature generally reduced both M_w_ and apparent viscosity, with only limited benefits in terms of yield under most conditions. This illustrates the classical compromise encountered during pectin extraction, as conditions that facilitate the solubilization of pectic material from the cell wall may, when sufficiently severe, simultaneously promote modification or depolymerization of the recovered polymer. This compromise, however, extends beyond structural changes, as recovering pectic fractions from GP in the present study without dephenolization or deproteinization steps also resulted in the recovery of other constituents from the GP matrix, meaning that the gravimetrically determined yield, which accounts for all material precipitated by ethanol, might mislead optimization if interpreted as pectin recovery alone. The condition giving the highest recovery, 7.88% with citric acid at pH 3.0 and 55 °C, cannot therefore be equated with the condition recovering the most pectin, making yield alone an insufficient criterion for selecting an extraction protocol. More broadly, extraction method and severity are now recognized as major determinants of pectin molecular weight, esterification and subsequent functionality, rather than merely of extraction efficiency.

Regarding acid type, the factorial analysis revealed a significant effect on the physicochemical properties of the extracted fractions, with citric acid consistently yielding low-methoxyl pectic fractions under the conditions investigated, whereas high-methoxyl pectic fractions were also recovered from the same GP material under selected conditions. However, these differences cannot be reduced to a simple distinction between an organic and a mineral acid, since the significant acid × pH × temperature interaction indicates that the influence of acid identity depended on the accompanying pH and temperature, jointly determining DE, molecular size and viscosity. The present extraction design therefore demonstrates that winemaking residue can provide pectic materials with substantially different physicochemical profiles simply by adjusting the extraction environment, without requiring different raw materials. However, beyond the variables considered here, other process parameters, including the solid-to-liquid ratio, sonication time and ultrasonic power, as well as the duration of the subsequent maceration step, were maintained constant and may also influence extraction efficiency and the characteristics of the recovered fractions. Incorporating these parameters into a broader multivariable optimization could therefore provide further refinement of the extraction process.

The characteristics of the starting grape pomace should also be considered when interpreting the present results. The industrial material consisted of a blend of Merlot, Syrah, Grenache, and Tempranillo residues, but the exact varietal proportions and detailed vintage and processing-history information were not available. Moreover, a complete compositional characterization of the starting material, including its initial protein, phenolic, uronic-acid, and carbohydrate composition, was not performed. Because grape-pomace composition can vary with cultivar, vintage, and winemaking conditions, these factors may influence extraction selectivity and the characteristics of the recovered pectic fractions. The present results should therefore be interpreted primarily for the specific grape-pomace batch investigated, and confirmation using grape pomaces of different origins and compositions would be valuable to assess the broader generalizability of the extraction trends identified here.

Interestingly, although the recovery of phenolic compounds and measurable amounts of protein alongside the pectic material contributes to reduced pectin purity, it can also be viewed as an opportunity to obtain a fraction with additional functional attributes. In the present study, the phenolic content and measurable ABTS scavenging activity support the potential value of recovering these constituents together through a single extraction process, which could broaden the potential applications of pectic fractions derived from GP, particularly where both the properties of the polysaccharide material and antioxidant activity are desirable. However, the extent to which these structural and compositional differences translate into properties such as gelation, emulsification, or film-forming behavior remains a limitation of the current study, and further technological evaluation is required.

## 4. Conclusions

This study demonstrated that acid type, pH and temperature jointly influenced the recovery and characteristics of pectic fractions obtained from grape pomace. Significant interactions among these extraction variables were observed for several responses, showing that their effects cannot be considered independently. Extraction yield ranged from 1.12 to 7.88%, while degree of esterification varied from 14.89 to 63.63%. Citric acid consistently favored low-methoxyl fractions, whereas HCl at pH 1.5 allowed the recovery of high-methoxyl fractions, highlighting the possibility of controlling esterification through the extraction conditions. In contrast, AUA remained within a comparatively narrow range of 63.27–69.24%, close to that of commercial citrus pectin (67.21%), suggesting that the extraction conditions affected DE more markedly than AUA.

A clear compromise was also observed between material recovery and preservation of macromolecular characteristics. Conditions favoring the highest yield did not necessarily produce the highest molecular weight or apparent viscosity, while increasing temperature generally promoted decreases in both M_w_ and viscosity. The recovered fractions also contained measurable amounts of phenolic compounds and exhibited ABTS radical-scavenging activity, indicating that phenolic constituents were co-recovered together with the pectic material and that their retention was strongly dependent on the extraction environment. Overall, the results show that the combined adjustment of acid type, pH and temperature provides a practical means of tailoring grape-pomace pectic fractions with distinct esterification, molecular, rheological and antioxidant profiles. These findings provide a basis for the subsequent selection and evaluation of specific fractions according to the functional properties required for future applications.

## Figures and Tables

**Figure 1:**
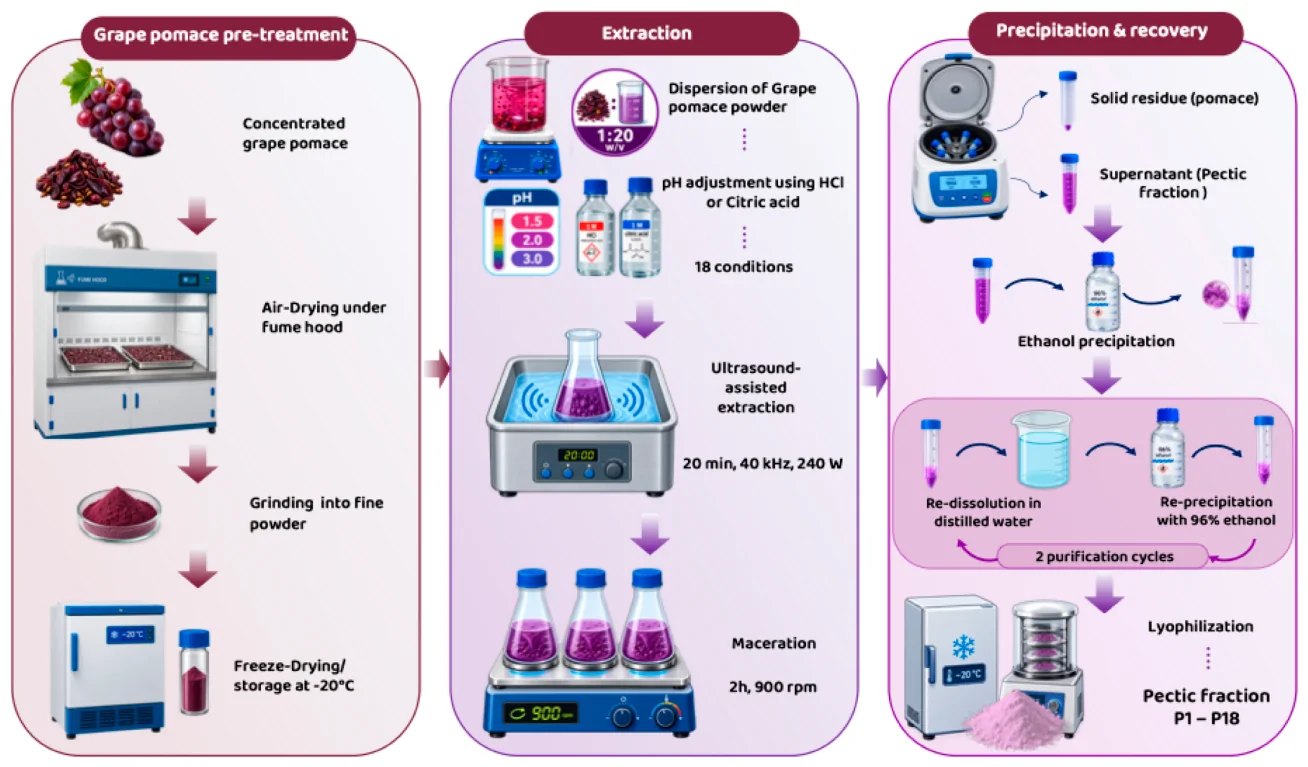
Schematic representation of grape pomace pretreatment and pectic fraction extraction by sequential ultrasound-assisted extraction and dynamic maceration.

**Figure 2:**
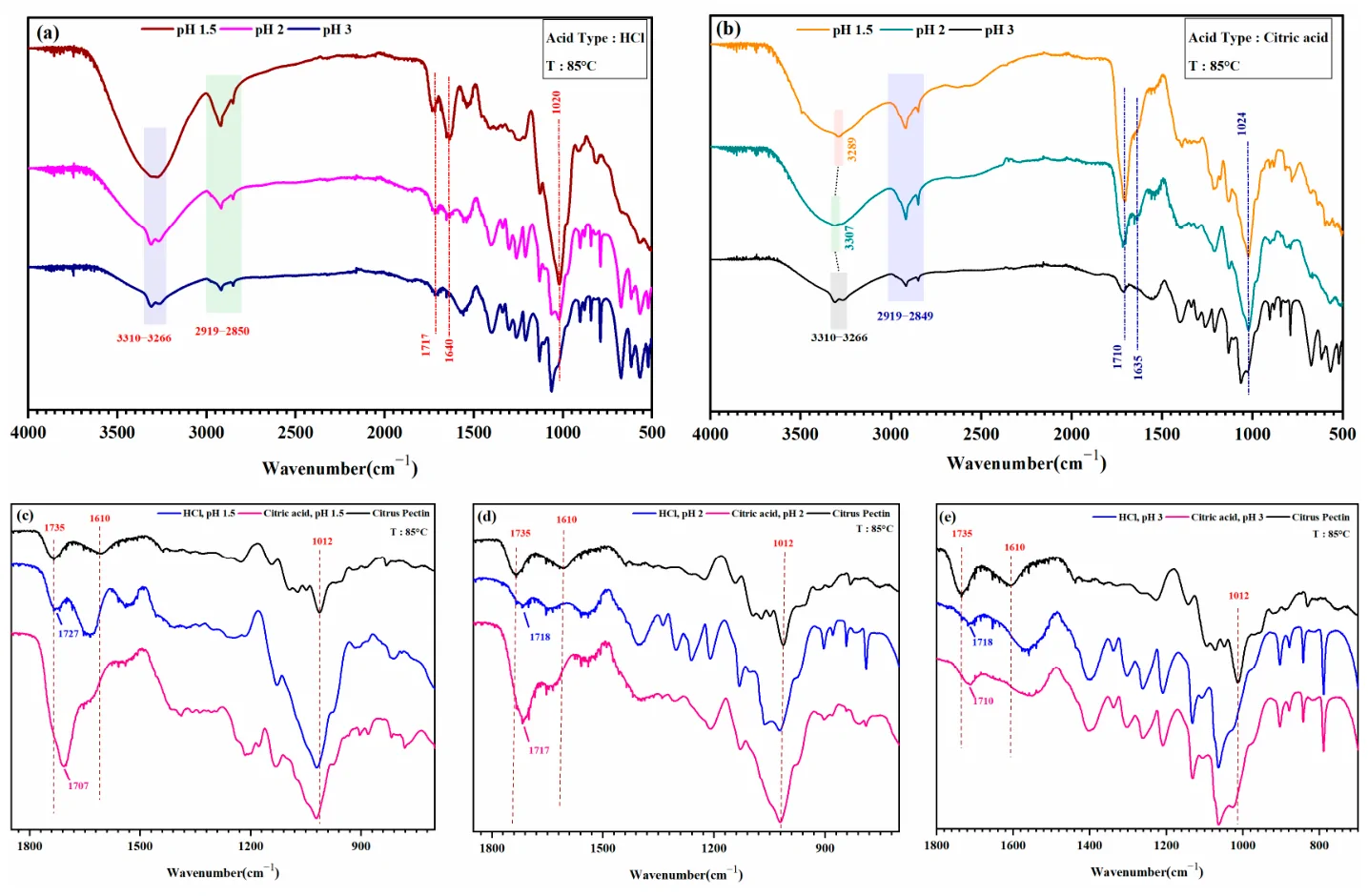
ATR-FTIR spectra of the pectic fractions extracted from grape pomace at 85 °C. (**a**) HCl and (**b**) citric acid, each at pH 1.5, 2.0 and 3.0; (**c**–**e**) comparison of the HCl- and citric acid-extracted pectic fractions with commercial citrus pectin at pH 1.5 (**c**), pH 2.0 (**d**) and pH 3.0 (**e**), shown over the 1800–800 cm^−1^ region.

**Figure 3:**
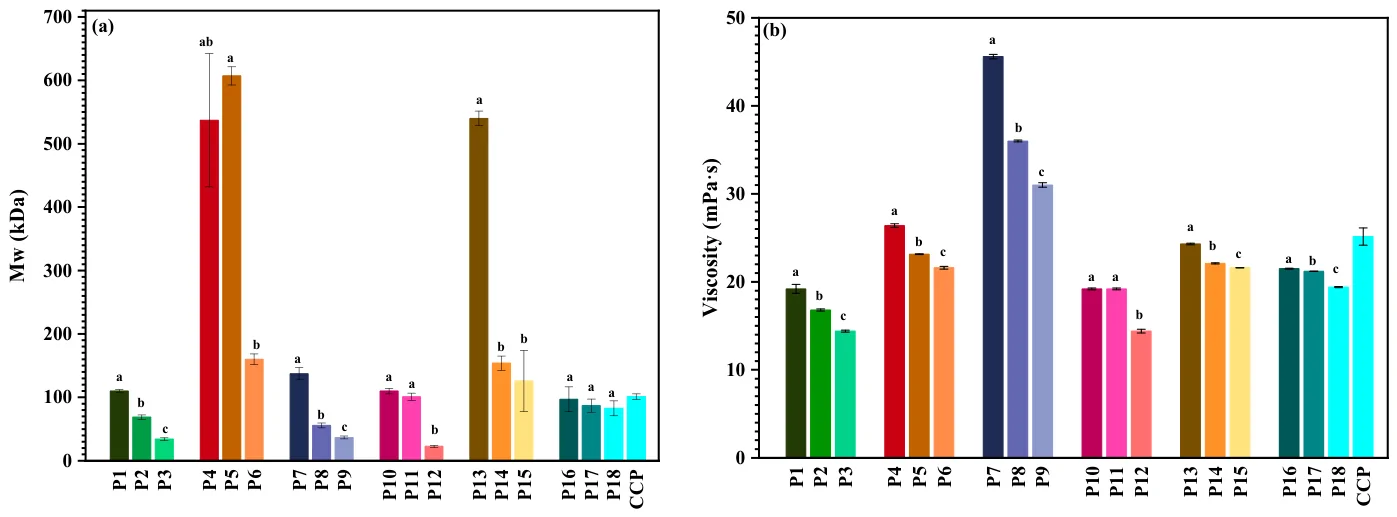
(**a**) Molecular weight (M_w_, kDa) and (**b**) apparent viscosity (mPa·s) of the pectic fractions extracted from grape pomace under the 18 conditions tested (P1–P18), with CCP included as a reference. Values are means of triplicate measurements. Different lowercase letters indicate significant differences among extraction temperatures within the same acid and pH condition (*p* < 0.05).

**Figure 4:**
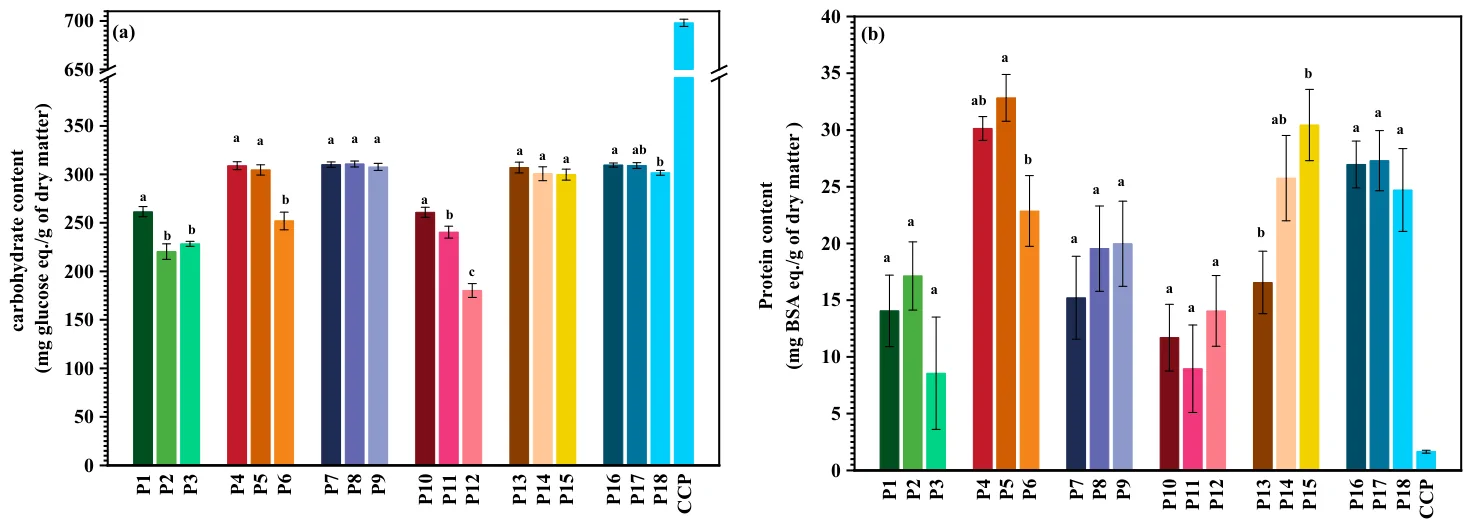
Quantification of the carbohydrate content and protein content in the extracted samples and in CCP (**a**) Carbohydrate content expressed as mg glucose eq./g of dry matter; (**b**) protein content expressed as mg BSA eq./g of dry matter of lyophilized recovered pectic fraction. Different lowercase letters indicate significant differences among extraction temperatures within the same acid and pH condition (*p* < 0.05).

**Table 1:** Three-way ANOVA for the effects of acid type, extraction pH, and temperature on extraction yield and the physicochemical characteristics of the extracted pectic fractions.

Source of Variation	Yield	DE	MeO	AUA	Mw	Apparent Viscosity	Total Carbohydrate Content	Protein Content	TPC	IC_50_
Acid	***	***	***	***	***	***	ns	ns	***	***
pH	***	***	***	ns	***	***	***	***	***	***
Temperature	***	***	***	***	***	***	***	*	***	***
Acid × pH	ns	***	***	***	***	***	***	***	***	***
Acid × Temperature	*	***	***	**	***	***	ns	***	***	***
pH × Temperature	***	***	***	***	***	***	***	ns	***	***
Acid × pH × Temperature	***	***	***	ns	***	***	***	***	***	***

ns, not significant; * *p* < 0.05; ** *p* < 0.01; *** *p* < 0.001.

**Table 2:** Summary of the experimental conditions applied for extraction of pectic fractions and the corresponding extraction yield (%). Different lowercase letters indicate significant differences among extraction temperatures within the same acid and pH condition (*p* < 0.05).

Sample	Acid	Temperature (°C)	pH	Yield (%/Dry Basis)
P1	Citric acid	55	3	7.88 ± 0.15a
P2	Citric acid	65		6.08 ± 0.10b
P3	Citric acid	85		6.20 ± 0.08b
P4	Citric acid	55	2	5.24 ± 1.55a
P5	Citric acid	65		2.52 ± 0.05a
P6	Citric acid	85		2.12 ± 0.65a
P7	Citric acid	55	1.5	2.00 ± 0.11b
P8	Citric acid	65		3.24 ± 0.15a
P9	Citric acid	85		3.20 ± 0.20a
P10	HCl	55	3	6.96 ± 0.71a
P11	HCl	65		5.64 ± 0.50a
P12	HCl	85		5.68 ± 0.25a
P13	HCl	55	2	2.80 ± 0.19a
P14	HCl	65		1.12 ± 0.67a
P15	HCl	85		2.76 ± 0.12a
P16	HCl	55	1.5	1.52 ± 0.05b
P17	HCl	65		1.32 ± 0.08b
P18	HCl	85		1.80 ± 0.10a

**Table 3:** Degree of esterification (DE), methoxyl content (MeO), and anhydrouronic acid content (AUA) of the pectic fractions extracted from grape pomace under different acid type, pH, and temperature conditions, compared with commercial citrus pectin (CCP) used as an external reference. Values are expressed as mean ± SD. Different lowercase letters within the same column indicate significant differences among extraction temperatures within the same acid and pH condition (*p* < 0.05).

Sample	Acid	pH	T (°C)	DE (%)	MeO (%)	AUA (%)
P1	Citric acid	3	55	30.00 ± 0.50a	3.34 ± 0.15a	63.27 ± 1.65b
P2	Citric acid	3	65	30.00 ± 0.60a	3.61 ± 0.50a	68.40 ± 1.05a
P3	Citric acid	3	85	29.00 ± 0.80a	3.54 ± 0.10a	69.24 ± 0.95a
P4	Citric acid	2	55	22.00 ± 0.75b	2.53 ± 0.05b	65.24 ± 0.85a
P5	Citric acid	2	65	23.00 ± 0.50b	2.64 ± 0.10b	65.24 ± 0.95a
P6	Citric acid	2	85	30.00 ± 1.05a	3.55 ± 0.11a	67.09 ± 1.05a
P7	Citric acid	1.5	55	14.89 ± 1.57c	1.66 ± 0.25c	63.27 ± 0.51a
P8	Citric acid	1.5	65	34.48 ± 2.08b	3.95 ± 0.35b	64.98 ± 1.12a
P9	Citric acid	1.5	85	43.47 ± 1.75a	5.00 ± 0.19a	65.31 ± 1.56a
P10	HCl	3	55	23.52 ± 0.75b	2.69 ± 0.08c	64.98 ± 0.98a
P11	HCl	3	65	25.16 ± 0.65b	3.01 ± 0.10b	67.82 ± 1.65a
P12	HCl	3	85	30.76 ± 0.50a	3.61 ± 0.06a	66.69 ± 1.01a
P13	HCl	2	55	25.00 ± 0.75c	2.94 ± 0.20b	66.82 ± 0.98a
P14	HCl	2	65	28.57 ± 1.05b	3.36 ± 0.10b	66.69 ± 0.57a
P15	HCl	2	85	33.33 ± 1.75a	4.02 ± 0.18a	68.41 ± 0.75a
P16	HCl	1.5	55	57.14 ± 2.06b	6.83 ± 0.23b	67.82 ± 1.19a
P17	HCl	1.5	65	63.63 ± 0.65a	7.65 ± 0.15a	68.24 ± 1.23a
P18	HCl	1.5	85	63.26 ± 0.70a	7.35 ± 0.10ab	65.98 ± 1.65a
CCP	---	---	---	72.95 ± 1.12	8.64 ± 0.16	67.21 ± 0.63

**Table 4:** CIE L*a*b* color coordinates and total color difference of the pectic fractions extracted from grape pomace with citric acid or HCl at pH 1.5, 2.0 and 3.0 and at 55, 65 and 85 °C (samples P1–P18), as well as of CCP. L* denotes lightness, a* the red–green coordinate and b* the yellow–blue coordinate; the total color difference (ΔE*ab) was calculated relative to the empty transparent Petri dish used as control, according to Equation (6). Visual color represents the approximate color appearance of each sample based on its measured CIELAB coordinate. Values are means ± standard deviation.

Sample	Acid Type	pH	T (°C)	L*	a*	b*	ΔE*ab	Visual Color
P1	Citric acid	3	55	73.28 ± 0.01	9.15 ± 0.00	5.93 ± 0.00	37.85 ± 0.01	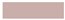
P2	Citric acid		65	69.72 ± 0.01	9.56 ± 0.00	6.62 ± 0.01	34.74 ± 0.01	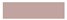
P3	Citric acid		85	63.32 ± 0.00	10.82 ± 0.00	3.50 ± 0.00	28.62 ± 0.00	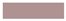
P4	Citric acid	2	55	61.48 ± 0.00	11.79 ± 0.00	6.56 ± 0.00	28.06 ± 0.00	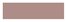
P5	Citric acid		65	62.03 ± 0.01	14.41 ± 0.00	5.93 ± 0.01	29.56 ± 0.01	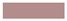
P6	Citric acid		85	61.96 ± 0.28	14.18 ± 0.16	4.65 ± 0.10	29.09 ± 0.14	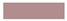
P7	Citric acid	1.5	55	51.80 ± 0.06	14.65 ± 0.03	3.02 ± 0.01	21.11 ± 0.06	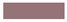
P8	Citric acid		65	51.36 ± 0.06	15.09 ± 0.05	3.25 ± 0.02	21.17 ± 0.08	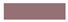
P9	Citric acid		85	56.55 ± 0.01	14.99 ± 0.01	4.24 ± 0.01	25.06 ± 0.01	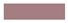
P10	HCl	3	55	70.93 ± 0.00	9.36 ± 0.01	5.98 ± 0.01	35.69 ± 0.00	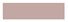
P11	HCl		65	62.09 ± 0.00	12.49 ± 0.01	0.58 ± 0.00	27.87 ± 0.01	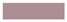
P12	HCl		85	66.94 ± 0.01	9.83 ± 0.01	5.09 ± 0.00	31.90 ± 0.00	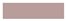
P13	HCl	2	55	61.08 ± 0.00	15.70 ± 0.01	2.59 ± 0.01	28.80 ± 0.00	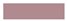
P14	HCl		65	60.82 ± 0.00	13.89 ± 0.01	4.87 ± 0.00	28.04 ± 0.00	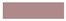
P15	HCl		85	64.75 ± 0.01	12.60 ± 0.01	4.81 ± 0.01	30.85 ± 0.01	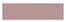
P16	HCl	1.5	55	53.61 ± 0.00	17.45± 0.01	0.80± 0.01	24.03 ± 0.00	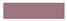
P17	HCl		65	66.31 ± 0.01	16.55 ± 0.00	1.66 ± 0.00	33.58 ± 0.00	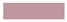
P18	HCl		85	66.17 ± 0.00	17.43 ± 0.00	0.43 ± 0.01	33.81 ± 0.01	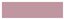
CCP				76.30 ± 0.01	3.65 ± 0.00	15.10 ± 0.01	39.87 ± 0.01	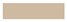

**Table 5:** Radical-scavenging activity, expressed as IC_50_, and total phenolic content (TPC) of the pectic fraction extracted from grape pomace with citric acid or HCl at pH 1.5, 2.0 and 3.0 and at 55, 65 and 85 °C (samples P1–P18); values for commercial citrus pectin (CCP) are shown for comparison. Values are means ± standard deviation. Different lowercase letters indicate significant differences among extraction temperatures within the same acid and pH condition (*p* < 0.05).

Sample	Acid Type	pH	T (°C)	IC_50_ (mg/mL)	TPC (mg GAE/g)
P1	Citric acid	3	55	8.02 ± 0.11b	11.24 ± 0.65ab
P2	Citric acid		65	7.57 ± 0.04c	13.33 ± 0.02a
P3	Citric acid		85	9.26 ± 0.02a	10.14 ± 0.09b
P4	Citric acid	2	55	10.38 ± 0.06a	9.50 ± 0.24b
P5	Citric acid		65	7.59 ± 0.11c	14.29 ± 0.35a
P6	Citric acid		85	7.98 ± 0.02b	14.16 ± 0.33a
P7	Citric acid	1.5	55	9.39 ± 0.17b	11.71 ± 0.45ab
P8	Citric acid		65	8.87 ± 0.10c	12.23 ± 0.34a
P9	Citric acid		85	9.91 ± 0.13a	10.79 ± 0.04b
P10	HCl	3	55	5.05 ± 0.07c	10.12 ± 0.30a
P11	HCl		65	5.47 ± 0.04b	11.14 ± 0.47a
P12	HCl		85	5.84 ± 0.03a	10.41 ± 0.07a
P13	HCl	2	55	6.82 ± 0.03a	10.62 ± 0.16ab
P14	HCl		65	7.39 ± 0.24a	10.57 ± 0.05b
P15	HCl		85	6.75 ± 0.04a	10.74 ± 0.05a
P16	HCl	1.5	55	4.26 ± 0.14b	9.77 ± 0.46b
P17	HCl		65	6.95 ± 0.17a	11.78 ± 0.12a
P18	HCl		85	6.84 ± 0.34a	12.08 ± 0.60a
CCP				10.12 ± 0.75	1.01 ± 0.15

## Data Availability

The original contributions presented in this study are included in the article. Further inquiries can be directed to the corresponding author.

## Supplementary Materials

The following supporting information can be downloaded at: https://www.mdpi.com/article/10.3390/polym18202465/s1, Figure S1. ATR-FTIR spectra of the pectic fractions recovered from grape pomace under the 18 extraction conditions, compared with commercial citrus pectin. (a) Citric acid, pH 3.0: P1, P2 and P3 obtained at 55, 65 and 85 °C, respectively; (b) citric acid, pH 2.0: P4, P5 and P6 at 55, 65 and 85 °C; (c) citric acid, pH 1.5: P7, P8 and P9 at 55, 65 and 85 °C; (d) hydrochloric acid, pH 3.0: P10, P11 and P12 at 55, 65 and 85 °C; (e) hydrochloric acid, pH 2.0: P13, P14 and P15 at 55, 65 and 85 °C; and (f) hydrochloric acid, pH 1.5: P16, P17 and P18 at 55, 65 and 85 °C. Spectra were recorded over the 4000–500 cm^−1^ range; Table S1. Full three-way factorial ANOVA results for the effects of acid type, extraction pH, and temperature on extraction yield and the physicochemical and functional characteristics of the pectic fractions extracted from grape pomace. F-values, *p*-values, and partial eta-squared (ηp^2^) are reported for the main effects and their two- and three-way interactions.
