# Supplementary material for: Ultrasound-Assisted Recovery of Pectic Fractions from Grape Pomace: Combined Effects of Acid Type, pH, and Temperature on Physicochemical and Antioxidant Properties
Source: Polymers (Basel). 2026 Oct 9;18(20):2465. doi: 10.3390/polym18202465 (PMC13709058; doi:10.3390/polym18202465)
Supplement: Supplementary file 1 [file polymers-18-02465-s001.zip › polymers-4598888-supplementary.pdf]

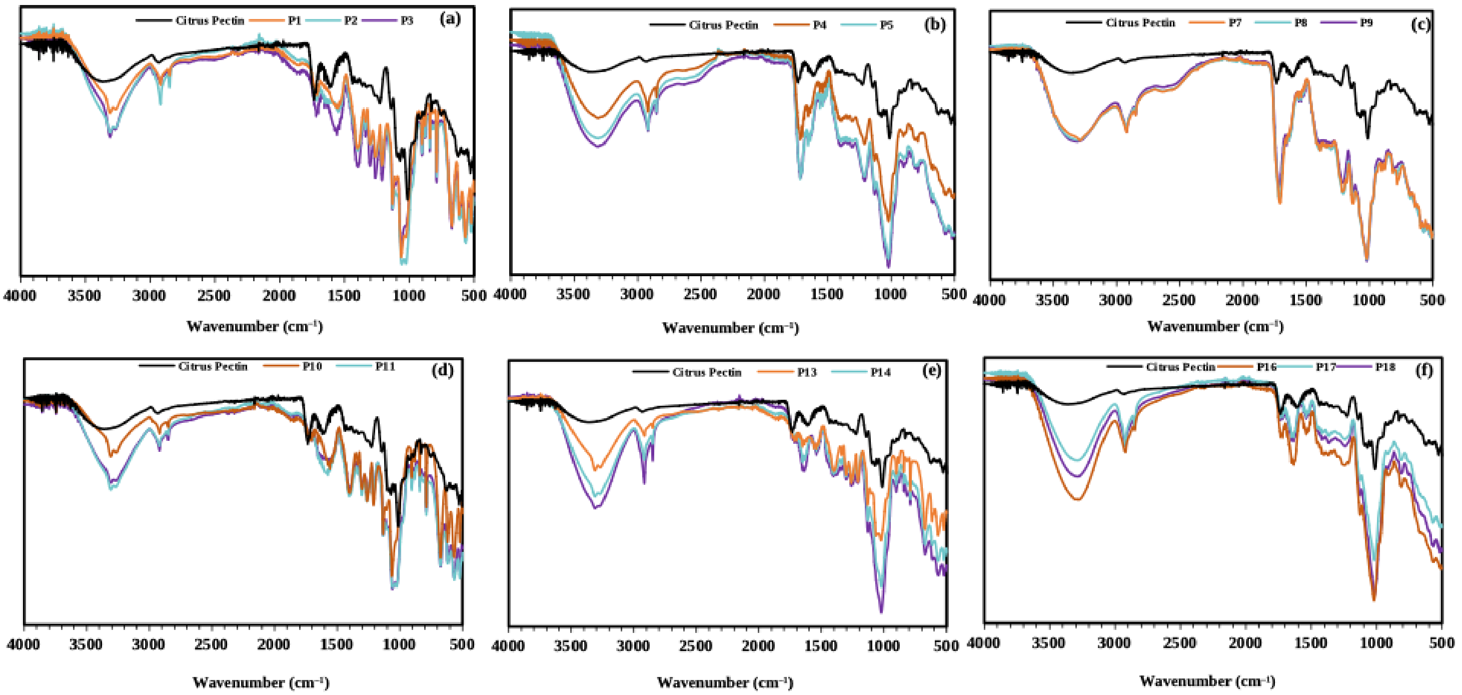

Figure S1. ATR-FTIR spectra of the pectic fractions recovered from grape pomace under the 18 extraction conditions, compared with commercial citrus pectin. (a) Citric acid, pH 3.0: P1, P2 and P3 obtained at 55, 65 and 85 °C, respectively; (b) citric acid, pH 2.0: P4, P5 and P6 at 55, 65 and 85 °C; (c) citric acid, pH 1.5: P7, P8 and P9 at 55, 65 and 85 °C; (d) hydrochloric acid, pH 3.0: P10, P11 and P12 at 55, 65 and 85 °C; (e) hydrochloric acid, pH 2.0: P13, P14 and P15 at 55, 65 and 85 °C; and (f) hydrochloric acid, pH 1.5: P16, P17 and P18 at 55, 65 and 85 °C. Spectra were recorded over the 4000–500 cm^{-1} range.

***Table S1**. Full three-way factorial ANOVA results for the effects of acid type, extraction pH, and temperature on extraction yield and the physicochemical and functional characteristics of the pectic fractions extracted from grape pomace. F-values, p-values, and partial eta-squared (ηp^2) are reported for the main effects and their two- and three-way interactions.*

**Response**	**Source of variation**	**df**	**F-value**	**p-value**	**Partial η²**	**Significance**
Yield	Acid	1, 36	55.325	8.817E-09	0.606	***
**Yield**	pH	2, 36	397.802	2.851E-25	0.957	***
**Yield**	Temperature	2, 36	23.471	2.989E-07	0.566	***
**Yield**	Acid × pH	2, 36	2.031	1.460E-01	0.101	ns
**Yield**	Acid × Temperature	2, 36	4.459	1.862E-02	0.199	*
**Yield**	pH × Temperature	4, 36	16.085	1.217E-07	0.641	***
**Yield**	Acid × pH × Temperature	4, 36	7.409	1.842E-04	0.452	***
**DE**	Acid	1, 36	1087.258	1.707E-28	0.968	***
**DE**	pH	2, 36	1554.720	1.135E-35	0.989	***
**DE**	Temperature	2, 36	307.396	2.352E-23	0.945	***
**DE**	Acid × pH	2, 36	1050.113	1.202E-32	0.983	***
**DE**	Acid × Temperature	2, 36	18.455	3.043E-06	0.506	***
**DE**	pH × Temperature	4, 36	74.421	6.689E-17	0.892	***
**DE**	Acid × pH × Temperature	4, 36	73.693	7.823E-17	0.891	***
MeO	Acid	1, 36	567.694	1.24×10^{-23}	0.940	***
**MeO**	pH	2, 36	712.096	1.13×10^{-29}	0.975	***
**MeO**	Temperature	2, 36	159.515	1.28×10^{-18}	0.899	***
**MeO**	Acid × pH	2, 36	532.192	1.84×10^{-27}	0.967	***
**MeO**	Acid × Temperature	2, 36	13.112	5.28×10^{-5}	0.421	***
**MeO**	pH × Temperature	4, 36	27.631	1.55×10^{-10}	0.754	***
**MeO**	Acid × pH × Temperature	4, 36	34.509	7.34×10^{-12}	0.793	***
**AUA**	Acid	1, 36	16.520	2.497E-04	0.315	***
**AUA**	pH	2, 36	2.467	9.904E-02	0.121	ns
**AUA**	Temperature	2, 36	14.569	2.315E-05	0.447	***
**AUA**	Acid × pH	2, 36	9.418	5.132E-04	0.343	***
**AUA**	Acid × Temperature	2, 36	6.726	3.298E-03	0.272	**
**AUA**	pH × Temperature	4, 36	7.512	1.656E-04	0.455	***
**AUA**	Acid × pH × Temperature	4, 36	1.467	2.325E-01	0.140	ns
**Mw**	Acid	1, 36	13.952	6.477E-04	0.279	***
**Mw**	pH	2, 36	209.632	1.461E-20	0.921	***
**Mw**	Temperature	2, 36	66.203	8.690E-13	0.786	***
**Mw**	Acid × pH	2, 36	20.180	1.324E-06	0.529	***
**Mw**	Acid × Temperature	2, 36	10.648	2.329E-04	0.372	***
**Mw**	pH × Temperature	4, 36	25.300	4.962E-10	0.738	***
**Mw**	Acid × pH × Temperature	4, 36	17.728	4.008E-08	0.663	***
**Apparent viscosity**	Acid	1, 36	14126.367	2.596E-48	0.997	***
**Apparent viscosity**	pH	2, 36	20621.518	8.518E-56	0.999	***
**Apparent viscosity**	Temperature	2, 36	4612.150	4.113E-44	0.996	***
**Apparent viscosity**	Acid × pH	2, 36	13637.240	1.444E-52	0.999	***
**Apparent viscosity**	Acid × Temperature	2, 36	1019.572	2.026E-32	0.983	***

**Apparent viscosity**	pH × Temperature	4, 36	368.492	1.150E-28	0.976	***
**Apparent viscosity**	Acid × pH × Temperature	4, 36	583.811	3.438E-32	0.985	***
**Total carbohydrate content**	Acid	1, 36	0.162	6.893E-01	0.004	ns
**Total carbohydrate content**	pH	2, 36	1073.252	8.171E-33	0.984	***
**Total carbohydrate content**	Temperature	2, 36	162.034	9.966E-19	0.900	***
**Total carbohydrate content**	Acid × pH	2, 36	23.765	2.631E-07	0.569	***
**Total carbohydrate content**	Acid × Temperature	2, 36	2.250	1.200E-01	0.111	ns
**Total carbohydrate content**	pH × Temperature	4, 36	38.988	1.287E-12	0.812	***
**Total carbohydrate content**	Acid × pH × Temperature	4, 36	54.739	8.194E-15	0.859	***
**Protein content**	Acid	1, 36	0.589	4.479E-01	0.016	ns
**Protein content**	pH	2, 36	88.658	1.233E-14	0.831	***
**Protein content**	Temperature	2, 36	3.490	4.117E-02	0.162	*
**Protein content**	Acid × pH	2, 36	18.292	3.299E-06	0.504	***
**Protein content**	Acid × Temperature	2, 36	8.993	6.797E-04	0.333	***
**Protein content**	pH × Temperature	4, 36	1.433	2.431E-01	0.137	ns
**Protein content**	Acid × pH × Temperature	4, 36	8.109	9.067E-05	0.474	***
**TPC**	Acid	1, 36	157.029	1.085E-14	0.813	***
**TPC**	pH	2, 36	14.057	3.079E-05	0.438	***
**TPC**	Temperature	2, 36	122.971	8.139E-17	0.872	***
**TPC**	Acid × pH	2, 36	28.029	4.575E-08	0.609	***
**TPC**	Acid × Temperature	2, 36	30.267	1.946E-08	0.627	***
**TPC**	pH × Temperature	4, 36	30.523	4.039E-11	0.772	***
**TPC**	Acid × pH × Temperature	4, 36	63.517	8.182E-16	0.876	***
**IC50**	Acid	1, 36	5260.957	1.268E-40	0.993	***
**IC50**	pH	2, 36	274.242	1.628E-22	0.938	***
**IC50**	Temperature	2, 36	68.906	4.921E-13	0.793	***
**IC50**	Acid × pH	2, 36	194.697	4.957E-20	0.915	***
**IC50**	Acid × Temperature	2, 36	392.603	3.575E-25	0.956	***
**IC50**	pH × Temperature	4, 36	210.957	1.887E-24	0.959	***
**IC50**	Acid × pH × Temperature	4, 36	61.226	1.452E-15	0.872	***

*df, degrees of freedom; ηp^2, partial eta-squared; ns, not significant; * $p < 0.05$; ** $p < 0.01$; *** $p < 0.001$. Error df = 36 for all responses.*
